# Saccade Dynamics in the Acute and Recovery Phase of Abducens Nerve Palsy

**DOI:** 10.18502/jovr.v19i4.14429

**Published:** 2024-12-31

**Authors:** Elissavet Kemanetzoglou, Klio Chatzistefanou, Nikolaos Smyrnis, Evangelia Kararizou, Evangelos Anagnostou

**Affiliations:** ^1^First Department of Neurology, National and Kapodistrian University of Athens, Eginition Hospital, School of Medicine, Athens, Greece; ^2^First Department of Ophthalmology, National and Kapodistrian University of Athens Gennimatas General Hospital, School of Medicine, Athens, Greece; ^3^Second Department of Psychiatry, National and Kapodistrian University of Athens, Attikon General Hospital, School of Medicine, Athens, Greece; ^5^Elissavet Kemanetzoglou: https://orcid.org/0000-0002-5800-6275; ^6^The manuscript has been submitted to medrxiv, as a preprint (https://doi.org/10.1101/2021.11.29.21267015)

**Keywords:** Abducens Nerve Palsy, Eye Movements, Saccade

## Abstract

**Purpose:**

To examine the natural adaptive course of ocular motor system in unilateral abducens nerve palsy while addressing the scarce literature on saccade dynamics and natural adaptation.

**Methods:**

Binocular horizontal eye movements were recorded from 18 healthy adults and 21 adults with unilateral abducens nerve palsy during the acute and chronic phases. Dynamics of the paretic and non-paretic eyes were compared, and the non-paretic eye dynamics were correlated with the respective prism diopters. Non-parametric tests were used for statistical comparisons.

**Results:**

The paretic eye, compared to the non-paretic eye, presented a slightly lower saccadic gain and velocity/amplitude ratio and a higher duration/amplitude ratio. The non-paretic eye, compared to healthy controls, showed consistent amplitude gain ( > 1) and a tendency for a higher duration/amplitude ratio. In the acute phase, when the non-paretic eye was covered, the paretic eye's amplitude ratio was lower and the duration/amplitude ratio decreased significantly. In the acute phase, a greater degree of esotropia in the paretic eye was associated with a lower amplitude gain and duration/amplitude ratio in the non-paretic eye.

**Conclusion:**

During adaptation in abducens nerve palsy, the saccade duration of the paretic eye increased, and a similar tendency was observed in the non-paretic eye. This finding likely reflects a change in the “pulse-step” pattern and may be related to plastic changes in central structures, such as the cerebellum, that support learning processes.

##  INTRODUCTION

Saccades are ballistic, short, voluntary, conjugate eye movements that abruptly change the focus and align the fovea with the object of interest,^[1]^ while multiple neural circuits engage in their control.^[2]^ Abducens nerve palsy is the most common ocular motor paralysis, which is characterized by horizontal diplopia in binocular vision when looking in the direction of the affected muscle (i.e., the respective lateral rectus).^[3]^ Abducens nerve palsy in adults is more often associated with vascular diseases, and complete or partial improvement usually occurs after months.^[4,5,6]^

Although saccades are the most studied ocular movements^[7]^ and an exceptional system for exploring neural motor control, literature is scarce about: (1) the effects of unilateral abducens nerve palsy on eye movement dynamics during saccades and (2) the variation of the endpoint of the paretic and non-paretic eye.^[8,9,10,11,12,13,14]^ Consequently, saccade adaptation during ocular palsy is still under investigation. Also, evaluating saccadic movements in abducens palsy can be a valuable clinical tool in locating the lesion and guiding the diagnostic approach, without subjecting the patient to expensive and time-consuming diagnostic tests.^[8,15,16,17,18]^

Despite the extensive literature on saccadic movements in both healthy controls and patients with central nervous system (CNS) disease, studies involving paralytic strabismus syndromes remain rare^[7,19,20,21]^. Our study aims to contribute to this area by investigating saccadic dynamics within this specific population of patients.

The purpose of this study was to investigate and record the maximum velocity, duration, and accuracy of horizontal saccades in individuals with unilateral abducens nerve palsy during the natural adaptive course of the ocular motor system in both the paretic and non-paretic eyes. We compared these saccade dynamics between the paretic and non-paretic eyes depending on whether the eye was open or covered (and thus fixating versus non-fixating, respectively). We also correlated non-paretic eye dynamics with the clinical deficit, measured in prism diopters, and delineated differences in saccade characteristics between patients with abducens nerve palsy and an age- and sex-matched control group (healthy eyes).

##  METHODS

A sample of 21 adults with microvascular abducens nerve palsy was recruited for this study. The microvascular etiology of abducens nerve palsy was based on the presence of diabetes mellitus or high blood pressure, negative brain imaging (MRI or CT scan), spontaneous palsy improvement no later than three months, and complete palsy recovery no later than six months after the onset of symptoms. Patients had to meet all four criteria in order to be eligible for the patient group. The control group was composed of 18 age- and sex-matched healthy adults who underwent the same tests as the patient group.

All participants provided written informed consent. The current study adhered to the principles of the Declaration of Helsinki and was approved by the Ethics Committee of the First Department of Neurology at the National and Kapodistrian University of Athens, Greece (approval number: A ΔA : 7EIN46 Ψ 8 N 2- ΨΓΘ ). The study took place at the Neuro-ophthalmology and Balance Research Laboratory of the Aeginiteion Hospital, which is affiliated with the aforementioned department.

Binocular horizontal saccades were recorded with an infrared corneal reflection device (IRIS Skalar, Delft, The Netherlands). Eye position signals were low-pass filtered with a cutoff frequency of 70 Hz, smoothed with a Savtizky Golay filter (second degree/eight samples), and digitized with a 14-bit analog-to-digital converter. Also, the signals were sampled at 500 Hz with a National Instruments external card. The digital signal was transmitted to a computer software developed in the LabVIEW environment (National Instrument, Austin, Texas). The digitized time series concerning the position of the right and left eyes were then further processed using custom MATLAB software developed specifically for the present study (MathWorks, Natick, Massachusetts).

**Table 1:** Comparative analysis of patient and control groups using Pearson's Chi-Square Test ( χ ² = 161, *P* = 0.144).


	**Patient group**	**Control group**
Age, y, mean (range)	56.48 (20–80)	58.28 (34–77)
25–75º percentile (in yrs)	47–70	51–67.5
Age, y, mean (SD)	17.48	10.65
Males–females, *n* (%)	57–43	39–61
	
	
SD, standard deviation; y, year; n, number		

**Table 2:** Demographic characteristics of the patient group.


**Patient group**		**Range**
Left eye palsy – Right eye palsy %	52–48%	
Mean time (in days) of palsy till the first recording session	30	2–82
Mean difference (in days) between first and second recording sessions	85	24–180
Mean diopters in the primary position, first recording session	25 ${\;}^{\Delta }$	5 ${\;}^{\Delta }$ –40 ${\;}^{\Delta }$
Mean diopters in the primary position, second recording session	0	0–10 ${\;}^{\Delta }$
	
	

**Table 3:** Comparative analysis of amplitude gain, velocity/amplitude ratio, and duration/amplitude ratio between healthy eye and non-paretic eye using Mann–Whitney test.


			**First session**		**Second session**	
	**OTD**	**Healthy eye**	**Non-paretic eye**	**Sig**	**Non-paretic eye**	**Sig**
**Amplitude gain**						
Eye open	5º	1.08 (0.93–1.23)	1.02 (0.97–1.16)	*P* > 0.05	1.04 (0.88–1.13)	*P* > 0.05
	10º	1.09 (1.00–1.25)	1.33 (1.02–1.43)	*P* > 0.05	1.06 (0.94–1.20)	*P* > 0.05
	15º	1.16 (1.05–1.29)	1.22 (1.07–1.72)	*P* > 0.05	1.16 (0.95–1.38)	*P* > 0.05
Eye covered	5º	1.00 (0.90–1.09)	0.98 (0.73–1.18)	*P* > 0.05	0.95 (0.88–1.19)	*P* > 0.05
	10º	1.12 (1.03–1.21)	1.08 (0.86–1.50)	*P* > 0.05	1.11 (0.89–1.23)	*P* > 0.05
	15º	0.99 (0.90–1.05)	1.15 (0.90–2.5)	*P* > 0.05	1.18 (0.99–1.42)	*P* > 0.05
**Velocity/Amplitude ratio**						
Eye open	5º	29.6 (25.7–33.3)	30.4 (26.7–32.9)	*P* > 0.05	30.2 (24.2–31.5)	*P* > 0.05
	10º	25.3 (22.2–29.0)	26.2 (22.4–29.2)	*P* > 0.05	26.3 (21.0–29.2)	*P* > 0.05
	15º	22.0 (18.2–25.9)	21.9 (16.7–25.9)	*P* > 0.05	22.3 (16.6–25.1)	*P* > 0.05
Eye covered	5º	29.3 (26.7–32.6)	26.1 (17.8–31.0)	*P* > 0.05	30.9 (26.6–37.0)	*P* > 0.05
	10º	24.2 (22.0–28.2)	21.8 (18.1–27.0)	*P* > 0.05	25.0 (19.9–27.0)	*P* > 0.05
	15º	22.7 (20.9–24.9)	20.0 (14.9–23.8)	*P* > 0.05	22.5 (16.7–25.1)	*P* > 0.05
**Duration/Amplitude ratio**						
Eye open	5º	13.8 (12.1–15.2)	13.4 (12.2–16.8)	*P* > 0.05	13.8 (11.1–15.0)	*P* > 0.05
	10º	8.6 (7.8–9.4)	9.5 (7.9–11.1)	*P* > 0.05	8.0 (7.3–10.0)	*P* > 0.05
	15º	7.0 (6.3–7.7)	7.5 (6.6–9.5)	*P* > 0.05	6.4 (5.5–8.1)	*P* > 0.05
Eye covered	5º	13.0 (11.5–13.9)	13.7 (11.3–17.0)	*P* > 0.05	11.8 (10.8–14.9)	*P* > 0.05
	10º	8.5 (7.7–9.1)	9.1 (6.8–11.3)	*P* > 0.05	8.9 (6.5–9.5)	*P* > 0.05
	15º	5.7 (5.2–6.1)	7.3 (5.1–13.1)	*P* > 0.05	7.0 (5.5–8.7)	*P* > 0.05
	
	
OTD, optical target distance; Sig, significance						

**Table 4:** Correlation between the angle of deviation and saccade dynamics of the non-paretic eye using the Spearman Correlation.


	**Open non-paretic eye**		**Covered non-paretic eye**	
	**First session**	**Second session**	**First session**	**Second session**
Amplitude gain	–0.244	0.033	–0,476	0.188
	*P* > 0.05	*P* > 0.05	* P * < 0.01	*P* > 0.05
Velocity/Amplitude ratio	–0.084	0.282	0.037	0.124
	*P* > 0.05	*P* > 0.05	*P* > 0.05	*P* > 0.05
Duration/Amplitude ratio	–0.080	–0.221	–0.404	–0.008
	*P* > 0.05	*P* > 0.05	*P* ** < ** 0.05	*P* > 0.05
	
	

**Table 5:** Comparison between paretic and non-paretic eye in the patient group using the Wilcoxon signed-rank test.


		**First session**			**Second session**		
	**OTD**	**Paretic eye**	**Non-paretic eye**	**Sig**	**Paretic eye**	**Non-paretic eye**	**Sig**
Amplitude gain				
Paretic eye open	5º	0.83	0.98	> 0.05	0.88	0.95	> 0.05
	(0.67–0.92)	(0.73–1.18)	(0.82–1.08)	(0.88–1.19)	
	10º	0.77	1.08	< 0.05	0.89	1.11	> 0.05
	(0.67–0.86)	(0.86–1.50)	(0.82–1.05)	(0.89–1.23)	
	15º	0.79	1.1	< 0.01	0.94	1.19	> 0.05
	(0.66–0.82)	(0.92–2.29)	(0.83–1.14)	(0.99–1.42)	
Paretic eye covered	5º	0.83	1.02	< 0.01	0.88	1.04	< 0.05
	(0.58–0.94)	(0.97–1.16)	(0.75–1.05)	(0.88–1.13)	
	10º	0.71	1.33	< 0.001	0.92	1.06	< 0.05
	(0.53–0.86)	(1.02–1.43)	(0.82–1.00)	(0.94–1.20)	
	15º	0.65	1.22	< 0.01	0.93	1.17	< 0.05
	(0.55–0.86)	(1.07–1.72)	(0.85–1.01)	(0.95–1.38)	
Velocity/Amplitude ratio				
Paretic eye open	5º	24.7	26.1	> 0.05	27.1	30.9	< 0.01
	(19.2–30.1)	(17. 8–31.0)	(23.9–29.7)	(26.6–37.0)	
	10º	20.8	21.8	> 0.05	23.3	25	> 0.05
	(14.6–25.0)	(18.1–27.0)	(19.7–26.0)	(19.9–27.0)	
	15º	19.1	20	> 0.05	18.8	22.5	> 0.05
	(16.6–22.4)	(15.7–21.6)	(14.8–21.4)	(16.7–25.1)	
Paretic eye covered	5º	26.6	30.4	< 0.05	28	30.2	> 0.05
	(20.8–29.6)	(26.7–32.9)	(24.9–30.8)	(24.2–31.5)	
	10º	26.1	26.2	> 0.05	23.9	26.3	> 0.05
	(18.1–28.7)	(22.4–29.2)	(20.9–26.0)	(21.0–29.2)	
	15º	22.5	21.9	> 0.05	21	22.3	> 0.05
	(19.5–25.2)	(16.7–25.9)	(18.4–22.8)	(16.6–25.1)	
Duration/Amplitude ratio				
Paretic eye open	5º	18.3	13.7	< 0.05	15	11.8	> 0.05
	(14.7–25.2)	(11.3–17.0)	(12.7–18.2)	(10.8–14.9)	
	10º	12.6	9.1	< 0.05	9.5	8.9	> 0.05
	(9.2–14.0)	(6.8–11.3)	(7.4–11.5)	(6.5–9.5)	
	15º	9.1	7.3	> 0.05	6.8	7	> 0.05
	(7.2–11.0)	(5.1–13.1)	(5.2–7.8)	(5.5–8.7)	
Paretic eye covered	5º	17.1	13.4	< 0.01	16.5	13.8	< 0.05
	(14.7–27.4)	(12.2–16.8)	(12.7–19.3)	(11.1–15.0)	
	10º	11.5	9.5	< 0.05	8.8	8	> 0.05
	(9.3–16.5)	(7.9–11.1)	(7.6–9.8)	(7.3–10.0)	
	15º	8.2	7.5	> 0.05	6.7	6.4	> 0.05
	(6.9–11.9)	(6.6–9.5)	(5.6–7.5)	(5.5–8.1)	
	
	
OTD, optical target distance; Sig, significance							

**Figure 1:**
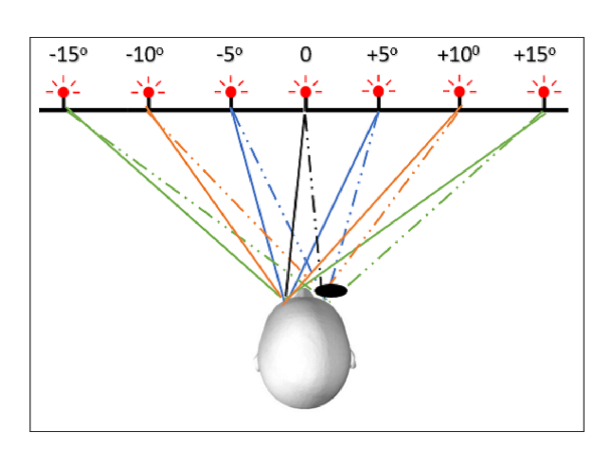
Schematic of the experimental setup used in the study for visually guided saccades (5º, 10º, and 15º) with right eye coverage (healthy eye in controls, paretic eye in cases of right eye palsy, non-paretic eye in cases of left eye palsy). Green lines depict 15º abduction, orange 10º abduction, and blue 5º abduction. Solid lines: viewing eye; dashed lines: covered eye. Simultaneous recording was performed in both eyes.

**Figure 2:**
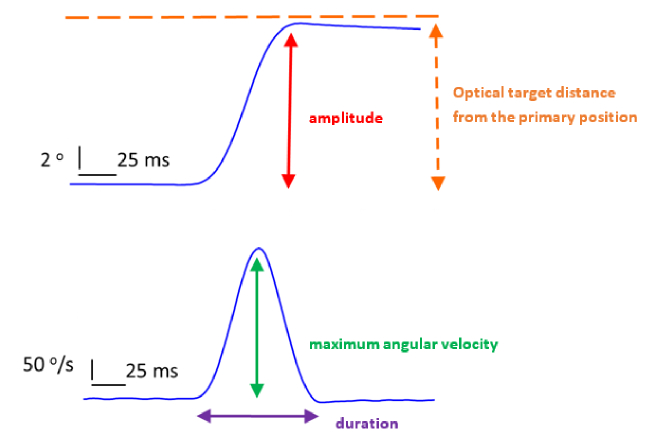
Example of a horizontal rightward 10º visually guided saccade recorded from a normal control participant with the eye open. The dynamic parameters are depicted in both the position (upper diagram) and velocity profile (lower diagram) of the eye. In each diagram, the horizontal axis represents time, while the vertical axis represents either position or velocity.

**Figure 3:**
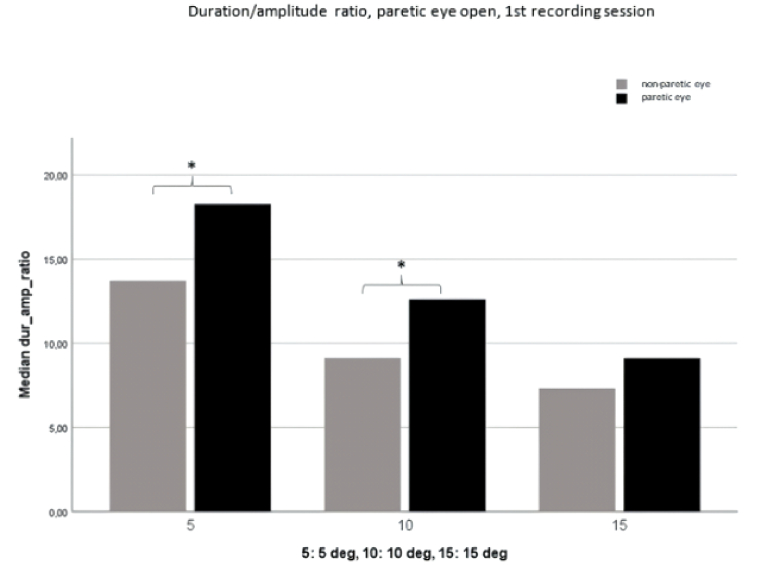
Duration/amplitude ratio with the paretic eye open. Comparison of the paretic eye with the non-paretic eye (first recording session). Significant differences are seen between 5º and 10º ( ${\;}^{*}$ = *P* < 0.05).

**Figure 4:**
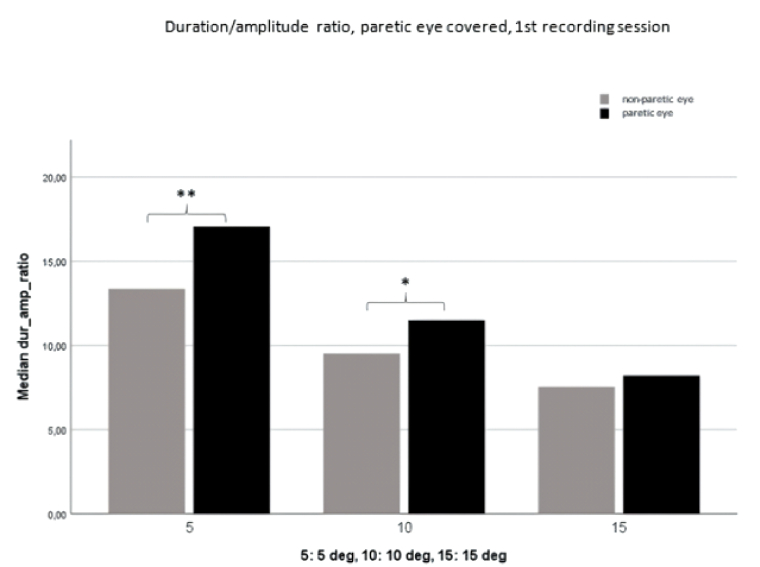
Duration/amplitude ratio with the paretic eye covered. Comparison of the paretic eye with the non-paretic eye (first recording session). Statistically significant differences are found at 5º (** = *P* < 0.01) and 10º (* = *P* < 0.05).

**Figure 5:**
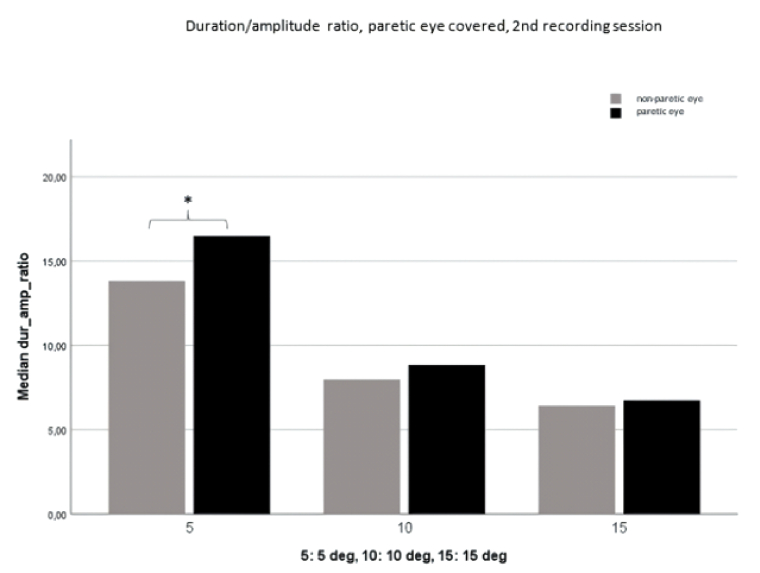
Duration/amplitude ratio with the paretic eye covered. Comparison of the paretic eye with the non-paretic eye (second recording session). The difference at 5º is statistically significant (* =* P* < 0.05).

**Figure 6:**
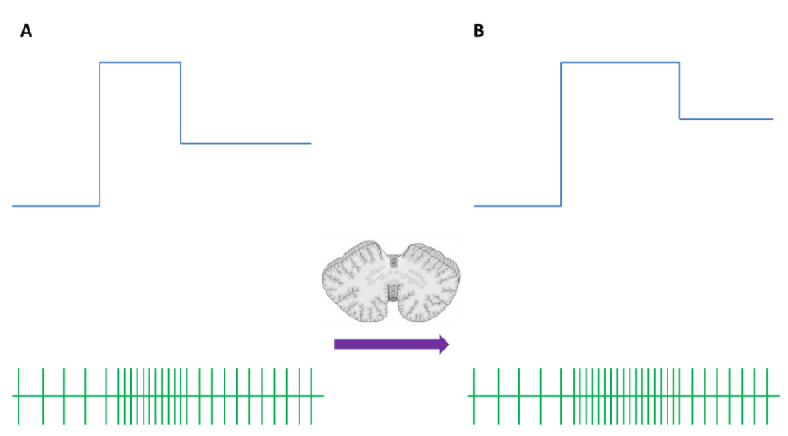
The upper panel schematically depicts the neuron discharge frequency of the abducens nucleus during a saccade. The “pulse-step” pattern is distinct. The lower panel schematically depicts the action potentials, from which the instantaneous discharge frequency of the upper panel is calculated. (A) Discharge pattern before and (B) after saccadic system adaptation. The difference lies in the increase in pulse width.

The horizontal primary saccades to the right and left were recorded binocularly (in both the paretic and non-paretic eyes) while the participant looked at fixed LED targets placed at ± 5º, ± 10º, and ± 15º eccentricities [Figure 1]. The recording device was calibrated at 10º on either side from the primary position of gaze. Then, the participant performed five rightward and five leftward saccades from the central fixation point. The same process was repeated after positioning the targets at 10º and then at 15º [Figure 2]. The eye cover permitted the movement and, thus, simultaneous recording of the non-viewing eye. Subsequently, the cover was moved to the contralateral eye and the whole procedure was repeated.

The purpose of the present study was to record the natural adaptive course of the ocular motor system following abducens nerve palsy, which was achieved on both the paretic and non-paretic eyes during fixation and non-fixation conditions. Although examining larger amplitude saccades (beyond 15º) would provide more data and possibly evaluate the performance limits of the paretic eye more vigorously, we preferred to use saccades up to 15º, as it is proven that this is the true natural range of the saccades in everyday conditions.^[22]^ Also, the use of fixed LED targets mimics natural ocular motor behavior better than targets flashing abruptly.^[23,24]^

The acute phase of abducens palsy was defined as the initial period, spanning 1 to 12 weeks, from the onset of diplopia until the symptoms stabilized without further progression. Diopters were then measured, and the saccades were recorded for the first time. Subsequently, the recovery phase, occurring between the fourth week and the sixth month, was delineated by the improvement of the patient's subjective symptom of diplopia and the objective evidence of fewer (or zero) diopters in the primary head position. During this stage, a second session was conducted to record saccades once again.

The saccade dynamics used for analysis were as follows:

1. The amplitude gain (amplitude/desired amplitude)

2. The peak velocity/amplitude ratio (max velocity/amplitude)

3. The duration/amplitude ratio (duration/amplitude)

Normalized fractions were used for statistical analysis while taking into account the so-called “main sequence” relation,^[25,26]^ which states that amplitude has a fixed relationship with duration and peak velocity of the saccade. These measurements reflect reality more accurately than do absolute values, a factor largely ignored in previous studies.

We analyzed the aforementioned dynamics in relation to the direction of attempted conjugate eye abduction in the field of action of the palsied lateral rectus muscle. In the case of right abducens nerve palsy, comparisons were made between right horizontal saccades, while in the case of left abducens nerve palsy, left horizontal saccades were compared.

SPSS version 21.0 (IBM, Armonk, New York) was used for statistical analysis with the level of significance set at 0.05. The Kolmogorov–Smirnov test, separately applied to each variable, demonstrated that amplitude gain, maximum velocity/amplitude ratio, and duration/amplitude ratio significantly deviated from the Gaussian distribution. Thus, non-parametric tests were used for statistical comparisons. The patient and the control groups were compared using the Pearson chi-square test. Dispersion of the healthy eye of the control group and the non-paretic eye of the patient group were compared using median and interquartile range respectively. On the other hand, the Mann–Whitney test was employed for comparisons between the healthy eye and the non-paretic eye. To examine whether the angle of deviation (prism diopters) was correlated with saccade dynamics of the non-paretic eye, we calculated Spearman's rank correlation coefficient (* ρ *). Finally, the Wilcoxon signed-rank test was used to draw comparisons between the paretic and the non-paretic eye.

##  RESULTS

The patient and control groups exhibited no significant differences and were age- and sex-matched [Table 1]. The demographic data of participants and their clinical data (including medical condition and prism diopters) were expressed using descriptive statistical methods [Table 2]. Table 3 summarizes the comparison between the healthy eye of the control group and the non-paretic eye in the patient group. We also investigated the correlation between the angle of deviation and saccade dynamics of the non-paretic eye [Table 4]. Table 5 presents a comparative analysis of the paretic and non-paretic eye in the patient group. The statistically significant results are highlighted in Tables 1–5.

Figure 3 shows that a significantly higher duration/amplitude ratio is evident during fixation of the paretic eye compared to the non-paretic eye; this is particularly notable at 5º and 10º during the initial recording session. Figures 4 and 5 further illustrate the prolonged duration of paretic eye saccades during fixation of the non-paretic eye, with statistically significant disparities observed at 5º and 10º during the first recording session and at 5º during the second recording session.

##  DISCUSSION

Since the final ocular motor pathway (abducens nerve – neuromuscular junction – lateral rectus muscle) is considered evenly intact in both the control and patient groups (for the non-paretic eye), any observed differences in saccade dynamics must be attributed to CNS adaptive changes.

In the present study, when the experimental condition forces the paretic eye to fixate on the target, the resulting retinal error can lead to an improvement in its saccadic performance. This is achieved by amplifying the central motor command and, in accordance with Hering's Law,^[27]^ increasing the range of motion of the non-paretic eye, especially when compared to healthy eyes. Additionally, the increase in the amplitude of the non-paretic eye, which should be perceived as the intensified effort of the central ocular motor system to compensate for the motor deficit of the paretic eye, is not greater than the initial clinical deficit that differed among patients.^[28,29]^ This observation suggests that the compensatory excess of the central command varies from person to person.^[30]^ This idiosyncratic ability to escalate muscle innervation seems to be influenced more by factors other than retinal error,^[31]^ such as adaptive changes in central structures (e.g., cerebellum), that serve learning processes and may vary among individuals. In conclusion, saccade duration and velocity show specific adaptive changes, proportional to the amplitude, in response to the contralateral eye palsy, but these changes do not reflect the magnitude of the clinical deficit.

Our findings, consistent with other studies demonstrated reduced velocity in the paretic eye compared to the non-paretic eye, although the difference was not statistically significant.^[32,33]^ Our results cannot be directly compared with those studies due to differences in the recording method, eye coverage conditions, clinical etiology of the palsy, and the calculation of angular velocity. Taking into account the "main sequence" relation,^[25,26]^ we expressed velocities as normalized fractions of maximal velocity/amplitude, which has not been considered in previous case reports. Similarly, small case series with abducens or oculomotor nerve palsy examined by conventional DC electro-ophthalmography have not attempted to normalize velocities.^[34,35]^

This study highlighted that the duration (normalized to the saccade amplitude) of both the paretic and non-paretic eyes was the primary dynamic parameter with the most significant change compared to healthy controls. This aligns with the findings of Daroff et al,^[1,22]^ which demonstrated that adaptive changes in motor parameters during successive recordings were primarily influenced by alterations in duration and amplitude rather than velocity. The increase in the duration of the saccades in the course of adaptive compensation of abducens nerve palsy was particularly evident in the second recording session, when the non-paretic eye performed orthometric saccades but with longer duration than that of healthy controls. This observation is consistent with the findings of Straube and Deubel.^[36]^ In that study, the “double step” technique was used in healthy individuals, introducing a residual retinal error that gradually adjusted the amplitude of the primary saccade by accommodating the initial shift. Straube and Deubel showed that participants who adjusted their saccades to increased amplitude of displacement also perceived an increase in the normalized duration of saccades.

Given that the motor command reaches the ocular muscles by a "pulse-step" neural discharge pattern, any adaptive change must be reflected as a change in this pattern. Our results did not show a clear increase in the maximum velocity of saccades in either of the recording sessions. On the other hand, the observed increase in duration indicated an increased amplitude of the pulse motor command [Figure 6]. This is consistent with the results reported by Daroff et al,^[1]^ where the increased duration of adapted saccades corresponded to a wide pulse width in the respective neural discharge.

It seems, therefore, that cerebellar learning processes begin immediately after an ocular motor nerve palsy, and in order to increase the range of saccadic movements and achieve accurate saccade endpoints, they enhance the pulse amplitude of the neuronal drive. As a result, the eye reaches the target slightly later, but with the correct amplitude, effectively reducing any retinal error.

Further research is needed to provide more insight into the plastic processes involved in the saccadic adaptation and the implications of CNS involvement in saccadic disorders.

##  Financial Support and Sponsorship

None.

##  Conflicts of Interest

None.
